# Survival from cancer of the lung in England and Wales up to 2001

**DOI:** 10.1038/sj.bjc.6604583

**Published:** 2008-09-23

**Authors:** B Rachet, M J Quinn, N Cooper, M P Coleman

**Affiliations:** 1 Cancer Research UK Cancer Survival Group, Non-Communicable Disease Epidemiology Unit, Department of Epidemiology and Population Health, London School of Hygiene and Tropical Medicine, Keppel Street, London WC1E 7HT, UK; 2 Social and Health Analysis and Reporting Division, Office for National Statistics (Room FG/114), 1 Myddelton Street, London EC1R 1UW, UK

Lung cancer is still the most common cancer in the world. Following the steady decline in incidence in men in England and Wales for over 20 years, it is now the second most common cancer in men (19 000 cases a year, 8% lifetime risk) after prostate cancer, and it causes 17 000 deaths a year. In women, the long-term increase in incidence has recently flattened off, but lung cancer is now the third most common cancer (13 000 cases a year, 4% lifetime risk) after cancers of the breast and large bowel, and it causes 11 500 deaths a year (Quinn *et al*, 2001; Office for National Statistics, 2005). Lung cancer is the commonest cause of death from cancer in both men and women.

Most lung cancers are caused by tobacco smoking, and incidence trends follow the trends of tobacco consumption, with an approximate 20-year time lag. In turn, mortality trends closely mirror the lung cancer incidence trends, with very little time lag, because survival has been so poor for so long. Lung cancer alone accounts for approximately 5% of all deaths in England and Wales. Screening has not been shown to reduce mortality. Survival from lung cancer has not improved much for decades, and even in European countries with the highest survival, 5-year relative survival remains less than 15% (Berrino *et al*, 1995b; Sant *et al*, 2003).

The most deprived group has a lung cancer incidence almost double that of the most affluent group, in both men and women (Quinn *et al*, 2001). Trends in incidence show a decline of approximately 20% since the late 1980s in men, and a plateau in the late 1990s for women: the trends are parallel, affecting all socioeconomic groups about equally (data not shown).

The survival analyses reported here relate to 392 000 patients diagnosed with a first, primary, invasive malignancy of the trachea, bronchus or lung in England and Wales during the period 1986–1999, who were followed up to 31 December 2001, some 79% of the 497 000 patients potentially eligible for inclusion. Of these, 1.5% were excluded because their vital status was unknown at 5 November 2002, when the data were extracted for analysis, and a further 16% because their survival was unknown (or zero): most of those were registered solely from a death certificate. A further 16 000 (3.2%) patients were excluded because they previously had cancer in another organ at some time since 1971.

Lung cancers assigned to the upper lobe comprised 23% of cases in the late 1980s, rising to 29% by the late 1990s, with a commensurate drop in the proportion with an unspecified lobe of origin, from 54 to 46%, suggesting improvement in the recording of pathology rather than changing patterns of disease. The lower lobe accounted for 12% of cases and the middle lobe 3%, with 8% assigned to the main bronchus. Bronchus and lung cancers predominate: those of the trachea accounted for only 0.3% of cases, unchanged since the 1970s. Approximately 28% were papillary or squamous carcinomas, 13% small cell or oat cell carcinomas and 12% adenocarcinomas, but 40% were poorly specified carcinomas.

## Survival trends

**Figure 1:**
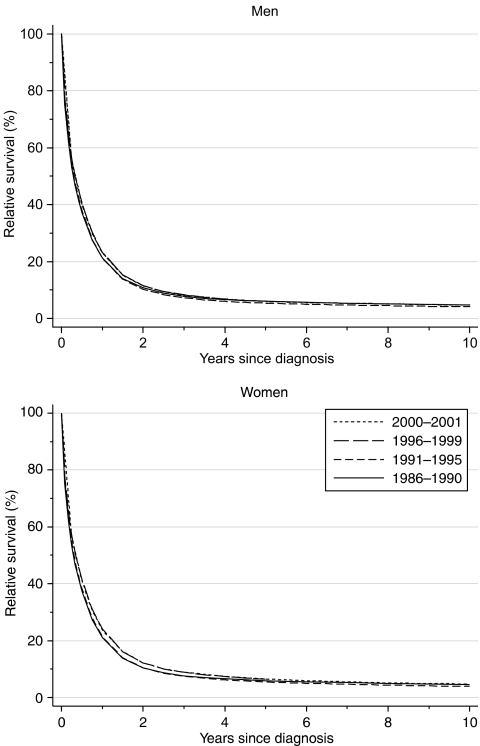
Relative survival (%) up to 10 years after diagnosis by sex and calendar period of diagnosis: England and Wales, adults (15–99 years) diagnosed during 1986–1999 and followed up to 2001. Survival estimated with cohort or complete approach (1986–1990, 1991–1995, 1996–1999) or hybrid approach (2000–2001) (see Rachet *et al*, 2008).

For patients diagnosed during 1996–1999, 1-year survival was 23–24%. This represents a small but statistically significant increase of 1.3% every 5 years since the late 1980s, after adjustment for deprivation (Table 1, Figure 1). Relative survival 5 or 10 years after diagnosis has remained extremely low, however, at 5–6% in both men and women. These survival rates are unchanged since the early 1970s (Coleman *et al*, 1999).

Predictions of survival, derived from patients' survival experience during the period 2000–2001 with hybrid analysis (Brenner and Rachet, 2004), do not suggest any imminent improvement in 1-year or 5-year survival.

## Deprivation

**Figure 2:**
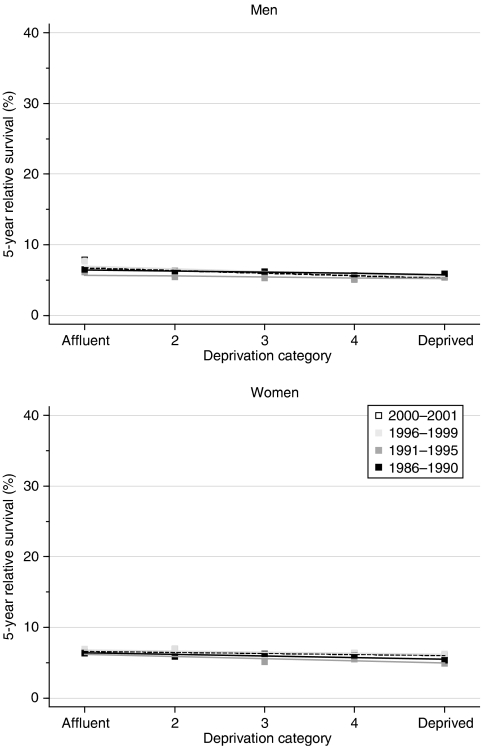
Trends in the deprivation gap in 5-year relative survival (%) by sex and calendar period of diagnosis: England and Wales, adults (15–99 years) diagnosed during 1986–1999 and followed up to 2001.

Despite the very low overall survival, a significant socioeconomic gradient in relative survival is evident in both sexes, with lower short-term and longer-term survival among the more deprived groups (Table 2). The fitted difference in 1-year survival between men in the most affluent and most deprived groups who were diagnosed during 1996–1999 was 3.4%. There is no evidence of a significant change in these deprivation gradients in survival (Table 2, Figure 2).

Short-term predictions from hybrid analysis do not suggest any imminent change in the socioeconomic gradient in survival.

## Comment

Survival from lung cancer remains desperately poor, at levels roughly similar to those for patients diagnosed 30 years ago (Coleman *et al*, 1999), although short-term survival may improve slightly in the near future.

Bias in these survival estimates should be considered. A substantial minority of cases (16%) had to be excluded from analysis because the only available information was from a death certificate, so their duration of survival was unknown. Approximately half these cases were from the south east (data not shown), and work elsewhere has shown that they would in fact have had shorter survival than the average for patients registered during life (Berrino *et al*, 1995a). In other words, the impact of excluding these death certificate only cases from the analyses would thus be to bias the survival estimates upwards, and not to reduce them.

One-year survival has nevertheless improved slightly but steadily during the 1990s. This may reflect the short-term efficacy of recent chemotherapy protocols for some morphological types of lung cancer. Another possible explanation is a trend towards earlier diagnosis. Information on the stage of disease at diagnosis was not available in these data. The average age of patients fell slightly during the years 1986–1999, possibly reflecting greater awareness of lung cancer and earlier diagnosis.

Both explanations may underlie the small but persistent deprivation gap in survival at 1 year after diagnosis. Compared with more deprived groups, affluent groups may have taken greater advantage of new treatments or lung cancer awareness campaigns, both of which may in turn have had an impact on short-term survival.

In the absence of substantial gains in lung cancer survival in England and Wales among patients diagnosed during 1986–1999, it should be noted that 1-year and 5-year survival in both countries was significantly lower than the average for patients diagnosed during 1990–1994 in the 22 countries contributing to the EUROCARE-3 study. After standardisation to a common age distribution in that study, 5-year lung cancer survival in England was 7.4% for men and 7.7% for women, compared with the European average values of 9.7 and 9.6%, respectively (Sant *et al*, 2003). In England, 1-year survival was 22.7% in both sexes, compared with European average values of 31.4 and 29.8% in men and women, respectively. In Wales, 1-year survival was 2–3% lower than in England, but 5-year survival was similar. Among western and northern European countries, the only country with lower lung cancer survival than England, Scotland and Wales was Denmark, where it has been attributed to late stage at diagnosis (Storm *et al*, 1999).

The persistent lack of improvement in lung cancer survival in England and Wales should be a cause for serious concern. Survival rates have improved in other European countries. Earlier diagnosis, enabling surgery and radiotherapy of curative intent in a higher proportion of cases, would appear the most likely approach to make progress.

## Figures and Tables

**Table 1:** Trends in relative survival (%) by sex, time since diagnosis and calendar period of diagnosis: England and Wales, adults (15–99 years) diagnosed during 1986–1999 and followed up to 2001

		**Calendar period of diagnosis^a^**									
		**1986–1990**		**1991–1995**		**1996–1999**		**Average change (%) every 5 years^b^**		**Prediction^c^ for patients diagnosed during 2000–2001**	
**Time since diagnosis**		**Survival (%)**	**95% CI**	**Survival (%)**	**95% CI**	**Survival (%)**	**95% CI**	**Survival (%)**	**95% CI**	**Survival (%)**	**95% CI**
1 year	Men	**21.3**	(21.1, 21.6)	**21.2**	(20.9, 21.5)	**23.2**	(22.9, 23.5)	**1.3** ^**^	(0.6, 1.9)	**23.0**	(22.6, 23.5)
	Women	**21.0**	(20.6, 21.4)	**21.5**	(21.1, 21.8)	**24.1**	(23.7, 24.5)	**1.3** ^**^	(0.4, 2.2)	**23.8**	(23.2, 24.4)
5 years	Men	**6.0**	(5.8, 6.2)	**5.4**	(5.2, 5.5)	**6.0**	(5.8, 6.3)	**0.1**	(−0.3, 0.5)	**6.0**	(5.7, 6.3)
	Women	**5.9**	(5.7, 6.2)	**5.5**	(5.3, 5.7)	**6.5**	(6.2, 6.8)	**0.1**	(−0.5, 0.7)	**6.5**	(6.1, 6.9)
10 years	Men	**4.7**	(4.5, 4.9)	**4.1**	(4.0, 4.3)			**−0.6**	(−1.3, 0.1)	**4.7**	(4.4, 5.0)
	Women	**4.5**	(4.3, 4.7)	**4.0**	(3.7, 4.2)			**0.2**	(−0.7, 1.2)	**4.8**	(4.4, 5.1)

CI=confidence interval.

a Survival estimated with cohort or complete approach (see Rachet *et al*, 2008).

b Mean absolute change (%) in survival every 5 years, adjusted for deprivation (see Rachet *et al*, 2008).

c Survival estimated with hybrid approach (see Rachet *et al*, 2008).

^**^P<0.01.

**Table 2:** Trends in the deprivation gap in relative survival (%) by sex, time since diagnosis and calendar period of diagnosis: England and Wales, adults (15–99 years) diagnosed during 1986–1999 and followed up to 2001

		**Calendar period of diagnosis^a^**									
		**1986–1990**		**1991–1995**		**1996–1999**		**Average change (%) every 5 years^b^**		**Prediction^c^ for patients diagnosed during 2000–2001**	
**Time since diagnosis**		**Deprivation gap (%)**	**95% CI**	**Deprivation gap (%)**	**95% CI**	**Deprivation gap (%)**	**95% CI**	**Deprivation gap (%)**	**95% CI**	**Deprivation gap (%)**	**95% CI**
1 year	Men	**−2.1** ^**^	(−2.9, −1.3)	**−1.1** ^**^	(−1.9, −0.3)	**−3.4** ^**^	(−4.4, −2.4)	**−0.4**	(−1.1, 0.2)	**−3.2** ^**^	(−4.6, −1.8)
	Women	**−1.3** ^*^	(−2.5, −0.1)	**−1.9** ^**^	(−3.0, −0.7)	**−0.7**	(−2.0, 0.6)	**0.3**	(−0.7, 1.2)	**−1.2**	(−3.1, 0.6)
5 years	Men	**−0.7** ^**^	(−1.2, −0.2)	**−0.5**	(−1.0, 0.0)	**−1.4** ^**^	(−2.2, −0.7)	**−0.3**	(−0.7, 0.2)	**−1.5** ^**^	(−2.3, −0.6)
	Women	**−0.9** ^*^	(−1.6, −0.1)	**−1.2** ^**^	(−1.9, −0.6)	**−0.6**	(−1.6, 0.3)	**0.0**	(−0.6, 0.7)	**−0.6**	(−1.7, 0.5)
10 years	Men	**−0.4**	(−0.9, 0.1)	**−0.4**	(−0.9, 0.2)			**0.0**	(−0.7, 0.8)	**−0.9** ^*^	(−1.8, −0.1)
	Women	**−0.7** ^*^	(−1.4, 0.0)	**−1.5** ^**^	(−2.2, −0.8)			**−0.8**	(−1.8, 0.1)	**−0.9**	(−1.9, 0.2)

CI=confidence interval.

a Survival estimated with cohort or complete approach (see Rachet *et al*, 2008).

b Mean absolute change (%) in the deprivation gap in survival every 5 years, adjusted for the underlying trend in survival (see Rachet *et al*, 2008).

c Survival estimated with hybrid approach (see Rachet *et al*, 2008).

^*^P<0.05; ^**^P<0.01.
